# Potential Maternal and Infant Outcomes from Coronavirus 2019-nCoV (SARS-CoV-2) Infecting Pregnant Women: Lessons from SARS, MERS, and Other Human Coronavirus Infections

**DOI:** 10.3390/v12020194

**Published:** 2020-02-10

**Authors:** David A. Schwartz, Ashley L. Graham

**Affiliations:** 1 Medical College of Georgia, Augusta University, Augusta, GA 30912, USA; 2 Department of Anthropology, University of Connecticut, Storrs, CT 06269, USA; ashley.graham@uconn.edu

**Keywords:** coronavirus, Middle East respiratory syndrome, severe acute respiratory syndrome, SARS-CoV, MERS-CoV, Wuhan coronavirus, 2019-nCoV, SARS-CoV-2, COVID-19, pregnancy, maternal mortality, maternal death, pregnancy complications, maternal morbidity, pneumonia, epidemic, emerging infection, China, Wuhan coronavirus outbreak

## Abstract

In early December 2019 a cluster of cases of pneumonia of unknown cause was identified in Wuhan, a city of 11 million persons in the People’s Republic of China. Further investigation revealed these cases to result from infection with a newly identified coronavirus, initially termed 2019-nCoV and subsequently SARS-CoV-2. The infection moved rapidly through China, spread to Thailand and Japan, extended into adjacent countries through infected persons travelling by air, eventually reaching multiple countries and continents. Similar to such other coronaviruses as those causing the Middle East respiratory syndrome (MERS) and severe acute respiratory syndrome (SARS), the new coronavirus was reported to spread via natural aerosols from human-to-human. In the early stages of this epidemic the case fatality rate is estimated to be approximately 2%, with the majority of deaths occurring in special populations. Unfortunately, there is limited experience with coronavirus infections during pregnancy, and it now appears certain that pregnant women have become infected during the present 2019-nCoV epidemic. In order to assess the potential of the Wuhan 2019-nCoV to cause maternal, fetal and neonatal morbidity and other poor obstetrical outcomes, this communication reviews the published data addressing the epidemiological and clinical effects of SARS, MERS, and other coronavirus infections on pregnant women and their infants. Recommendations are also made for the consideration of pregnant women in the design, clinical trials, and implementation of future 2019-nCoV vaccines.

## 1. Introduction

Coronaviruses are spherical, enveloped, and the largest of positive-strand RNA viruses. They have a wide host range, including birds, farm animals, pets, camels, and bats, in which they primarily cause respiratory and gastrointestinal disease. Belonging to the order *Nidovirales*, family *Coronaviridae*, and the subfamily *Orthocoronaviridae* there are four genera of coronaviruses—*Alphacoronavirus*, *Betacoronavirus*, *Deltacorona* virus, and *Gammacoronavirus* [1,2,3,4].

In humans, they are a cause of mild illnesses including the common colds occurring in children and adults, and were believed to be of modest medical importance. However, two zoonotic coronaviruses—including the severe acute respiratory syndrome coronavirus (SARS-CoV) and Middle East respiratory syndrome coronavirus (MERS-CoV)—can produce severe lower respiratory tract infections. Both the SARS-CoV and MERS-CoV have several features in common that are factors in producing nosocomial transmission, replication in the lower respiratory tract, and viral immunopathology. Both coronaviruses are zoonotic infections and constitute significant public health threats that have resulted in epidemics with significant loss of life [1,5,6]. When the SARS-CoV and MERS-CoV infect women who are pregnant, they can result in poor obstetric outcomes including maternal morbidity and death. There are currently no vaccines or specific treatments approved for coronavirus infections [2,6].

Prior to December 2019, there were a total of six coronavirus species that produced human infection: HCoV-229E and HCoV-NL63 belonging to the *Alphacoronaviru*s genus; and HCoV-OC43, HCoV-HKU1, MERS-CoV, and SARS-CoV, which belong to the *Betacoronavirus* genus [1,2]. As of December 2019, there are now seven species that infect humans.

As the newly identified novel coronavirus, termed 2019-nCoV and subsequently named SARS-CoV-2, spreads rapidly throughout China and across to other countries, researchers scramble to understand transmission dynamics, virulence, and pathogenicity. Given the rapidly progressive spread of this current 2019 novel coronavirus it is reasonable to expect that pregnant women have already become infected. The effect of 2019-nCoV during pregnancy is, at the present, unknown. This communication reviews the medical and clinical findings from coronavirus infections in pregnant women in order to anticipate how the newly discovered 2019-nCoV might affect maternal and infant morbidity and mortality.

## 2. The 2019 Coronavirus 2019-nCoV (SARS-CoV-2) Outbreak in Wuhan

**Figure 1:**
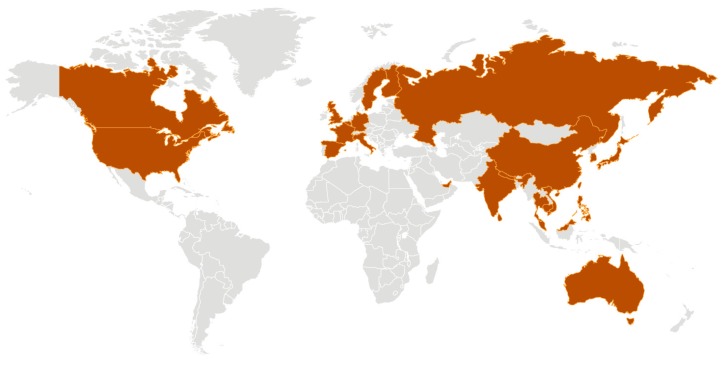
Distribution of countries with confirmed cases of the newly identified coronavirus 2019-nCoV (also termed SARS-CoV-2) infection as of 7 February 2020. Courtesy of the U.S. Centers for Disease Control and Prevention, Atlanta, GA.

In the beginning of December 2019, a cluster of persons with a pneumonia of unknown cause was identified in Wuhan, the capital of Hubei Province and a large city of approximately 11 million persons located in the central region of the People’s Republic of China [7,8]. Between 8 and 18 December 2019 there were 7 cases of pneumonia identified whose clinical features resembled that of a viral pneumonia. The outbreak was initially believed to be linked to the Wuhan Huanan (South China) Seafood Wholesale Market. This market, termed a “wet” market, sells a variety of seafood, cuts of meat, and both live and dead animals in over one thousand stalls in constant close contact; however, whether this market was the origin of the outbreak remains unknown [9]. On 31 December 2019, the Chinese Center for Disease Control and Prevention (China CDC) sent a rapid response team to Hubei to work alongside health personnel from the provincial and Wuhan city health departments to conduct an epidemiologic investigation. As the disease was spreading through secondary and tertiary cases, the World Health Organization (WHO) China Country Office was informed on 31 December 2019 of the occurrence of these cases of pneumonia of unknown etiology. During the period from 31 December 2019 to 3 January 2020, 44 patients with pneumonia of unknown etiology were reported by the Chinese authorities to the WHO. On 7 January 2020 investigators in China identified the etiological agent of the epidemic as a previously unknown coronavirus, and it was given the designation 2019-nCoV (for 2019 novel coronavirus) [8]. Analysis of the clinical features of 41 hospitalized patients with laboratory-confirmed 2019-nCoV infection revealed that 30 were men (73%); less than one-half had underlying co-morbid conditions (13; 32%) which included diabetes (8, 20%), hypertension (6, 15%), and cardiovascular disease (6; 15%); and the average age was 49.0 years old. The most common symptoms at the beginning of their illness included fever (40, 98%), cough (31, 76%), and fatigue or myalgia (18, 44%), sputum production (11, 28%), and headache (3, 8%) [10]. Among these 41 initial cases of 2019-nCoV infection there were 12 patients (32%) who developed acute respiratory distress syndrome (ARDS), 13 (32%) required intensive care and 6 (15%) died. During the first weeks of January the infection spread rapidly through China and extended to adjacent countries where cases began to appear—13 January in Thailand, 15 January in Japan, 20 January in the Republic of Korea, and Taiwan and the United States on 21 January [11]. Infected travelers, mostly via commercial air travel, are known to have been responsible for introducing the virus outside of Wuhan. The new coronavirus continued to spread throughout multiple countries and continents, and by 9 February 2020 the WHO reported 37,251 confirmed cases in China that resulted in 812 deaths, surpassing the number of deaths that occurred during the 2002–2003 SARS epidemic. An additional 307 cases of 2019-nCoV infection have occurred among 24 other countries outside of China [12]. (Figure 1) At the meeting of the Emergency Committee of the WHO on 30 January, the novel coronavirus 2019 epidemic was declared a Public Health Emergency of International Concern (PHEIC) [11,13].

This newly recognized coronavirus, producing a disease that has been termed COVID-19, is rapidly spreading throughout China, has crossed international borders to infect persons in neighboring countries, and humans infected by the virus are travelling via commercial airlines to other continents. It is certain that 2019-nCoV will infect women who are pregnant, leaving the question open as to whether the novel coronavirus will have a similar or different effect on them compared with SARS-CoV and MERS-CoV. In order to address the potential obstetrical outcomes of infection to both mother and infant, the present communication describes the current state of knowledge regarding the effects of other coronavirus infections in pregnancy. 

## 3. Pneumonia Occurring during Pregnancy

Pneumonia arising from any infectious etiology is an important cause of morbidity and mortality among pregnant women. It is the most prevalent non-obstetric infectious condition that occurs during pregnancy [14,15,16]. In one study pneumonia was the 3rd most common cause of indirect maternal death [17]. Approximately 25 percent of pregnant women who develop pneumonia will need to be hospitalized in critical care units and require ventilatory support [16]. Although bacterial pneumonia is a serious disease when it occurs in pregnant women, even when the agent(s) are susceptible to antibiotics, viral pneumonia has even higher levels of morbidity and mortality during pregnancy [18]. As with other infectious diseases, the normal maternal physiologic changes that accompany pregnancy—including altered cell-mediated immunity [19] and changes in pulmonary function—have been hypothesized to affect both susceptibility to and clinical severity of pneumonia [20,21,22]. This has been evident historically during previous epidemics. The case fatality rate (CFR) for pregnant women infected with influenza during the 1918–1919 pandemic was 27%—even higher when exposure occurred during the 3rd trimester and upwards of 50% if pneumonia supervened [23]. During the 1957–1958 Asian flu epidemic, 10% of all deaths occurred in pregnant women, and their CFR was twice as high as that of infected women who were not pregnant [24]. The most common adverse obstetrical outcomes associated with maternal pneumonias from all causes include premature rupture of membranes (PROM) and preterm labor (PTL), intrauterine fetal demise (IUFD), intrauterine growth restriction (IUGR), and neonatal death [14,15,16]. 

## 4. The 2002–2003 Severe Acute Respiratory Syndrome (SARS) Epidemic

The SARS epidemic began quietly at the turn of the 21st century. In November 2002, a cook in Guangdong Province, China, died from an unidentified illness. He had worked at a restaurant in which meat from wild animals was served. On 27 November 2002 Chinese-language media and internet reports were picked up by Canada’s Global Public Health Intelligence Network (GPHIN) that indicated a flu-like illness was occurring in China [25,26]. Unfortunately, the reports were not translated, and China failed to report the occurrence of this illness to the World Health Organization (WHO) until February 2003. The disease spread to other countries where it primarily infected healthcare workers. One of these was Dr. Carlo Urbani, a WHO physician investigating a patient with the new disease in Hanoi. He recognized that the pneumonia was probably caused by a new, highly infectious agent, and rapidly notified the WHO. He contracted the SARS-CoV while there, became febrile and later died after traveling to Thailand to attend a conference. On 12 March 2003, WHO issued a global alert regarding the disease that was occurring primarily among health care workers in Hanoi, Vietnam and Hong Kong. The disease continued to spread, and by 31 July 2003 there were 8422 probable cases, leading to 916 deaths in 29 countries, with the majority of cases occurring in mainland China and Hong Kong. Approximately 30% of infections occurred in healthcare workers. By the termination of the epidemic the global CFR was 11% [27].

## 5. SARS and Pregnancy

Although there were relatively few documented cases of SARS occurring during pregnancy, several case reports and small clinical studies have described the clinical effects in pregnant women and their infants. In reviewing these reports describing pregnant women with SARS in China it is possible, and perhaps even probable, that some of the same patients were included in more than one publication. However, even if this is the case, there is no doubt that SARS coronavirus infection was found to be associated with severe maternal illness, maternal death, and spontaneous abortion [19,28,29,30,31]. Martha Anker, an expert in statistics formerly with the WHO and the University of Massachusetts, estimated that more than 100 cases of SARS-CoV infection occurred in pregnant women, which warrants closer inspection [27].

The clinical outcomes among pregnant women with SARS in Hong Kong were worse than those occurring in infected women who were not pregnant [32]. Wong et al. [29] evaluated the obstetrical outcomes from a cohort of pregnant women who developed SARS in Hong Kong during the period of 1 February to 31 July 2003. Four of the 7 women (57%) that presented during the 1st trimester sustained spontaneous miscarriages, likely a result of the hypoxia that was caused by SARS-related acute respiratory distress. Among the 5 women who presented after 24 weeks gestation, 4 had preterm deliveries (80%).

A case-control study to determine the effects of SARS on pregnancy compared 10 pregnant and 40 non-pregnant women with the infection at the Princess Margaret Hospital in Hong Kong [27,33]. There were 3 deaths among the pregnant women with SARS (maternal mortality rate of 30%) and no deaths in the non-pregnant group of infected women (*P* = 0.006). Renal failure (*P* = 0.006) and disseminated intravascular coagulopathy (*P* = 0.006) developed more frequently in pregnant SARS patients when compared with the non-pregnant SARS group. Six pregnant women with SARS required admission to the intensive care unit (ICU) (60%) and 4 required endotracheal intubation (40%), compared with a 12.5% intubation rate (*P* = 0.065) and 17.5% ICU admission rate (*P* = 0.012) in the non-pregnant group.

Maxwell et al. [32] reported 7 pregnant women infected with SARS-CoV who were followed at a designated SARS unit—2 of the 7 died (CFR of 28%), and 4 (57%) required ICU hospitalization and mechanical ventilation. In contrast, the mortality rate was less than 10% and mechanical ventilation rate less than 20% among non-pregnant, age-matched counterparts who were not infected with SARS-CoV. Two women with SARS recovered and maintained their pregnancy but had infants with IUGR. Among the live newborn infants, none had clinical or laboratory evidence for SARS-CoV infection. The new mothers who had developed SARS were advised not to breastfeed to prevent possible vertical transmission of the virus.

Zhang et al. [34] described SARS-CoV infections in 5 primagravidas from Guangzhou, China at the height of the SARS epidemic. Two of the mothers became infected in the 2nd trimester, and 3 developed infection in the 3rd trimester. Two of the pregnant women had hospital-acquired SARS infections, and the other 3 were community-acquired. All 5 pregnant women had fever and abnormal chest radiographs; 4 had cough; 4 developed hypoalbuminemia; 3 had elevated alanine aminotransferase levels (ALT), 3 had chills or rigor, 2 had decreased lymphocytes, and 2 had decreased platelets. One pregnant woman required intensive care, but all recovered and there were no maternal deaths. The 5 infants were clinically evaluated, and none had evidence of SARS.

Two pregnant women with SARS were reported from the United States. In a detailed case report, Robertson et al. [35] described a 36-year-old pregnant woman with an intermittent cough of approximately 10 days duration and no fever. While travelling in Hong Kong during the 2003 epidemic, she was exposed at her hotel to a person subsequently known to be infected with SARS-CoV. At 19 weeks gestation she developed fever, anorexia, headache, increasing cough, weakness, and shortness of breath. Upon returning to the United States she was hospitalized with pneumonia. Obstetrical ultrasounds revealed a low-lying placenta (placenta previa) but were otherwise normal. Following her discharge home and clinical recovery, she was found to have antibodies to SARS-CoV. She underwent cesarean section at 38 weeks gestation because of the placenta previa and a healthy baby girl was delivered [35,36]. The placenta was interpreted as being normal. At 130 days post-maternal illness, maternal serum and whole blood, swabs from maternal nasopharynx and rectum, post-delivery placenta, umbilical cord blood, amniotic fluid, and breast milk were collected for analysis—no viral RNA was detected in specimens tested by reverse transcriptase polymerase chain reaction (RT-PCR). Antibodies to SARS-CoV were detected from maternal serum, umbilical cord blood, and breast milk by enzyme immunoassay (EIA) and indirect immunofluorescence assay. No clinical specimens (except for cord blood) were available for testing from the infant. The second case in the USA occurred in a 38-year-old woman who had travelled to Hong Kong at 7 weeks gestation where she was exposed to SARS-CoV in the same hotel as the aforementioned American woman [37]. Following her return to the United States, her husband developed the clinical onset of SARS, and 6 days later she became ill with fever, myalgia, chills, headache, coryza, and a productive cough with shortness of breath and wheezing. Following her hospitalization for SARS she recovered, serum samples taken on days 28 and 64 post-onset of illness were positive for antibodies to SARS-CoV by enzyme immunoassay and immunofluorescent assays. Her pregnancy continued and was unremarkable except for developing elevated glucose levels. A cesarean section that was performed at 36 weeks gestation due to preterm rupture of membranes and fetal distress resulted in a healthy baby boy. At the time of delivery, the mother’s serum samples were positive for antibodies to SARS-CoV, but samples taken of umbilical cord blood and placenta were negative. Breast milk sampled 12 and 30 days after delivery were also negative for SARS-CoV antibodies. Specimens evaluated from maternal blood, stool, and nasopharynx samples, as well as umbilical cord blood of the infant, were all negative for coronavirus RNA by RT-PCR. Neonatal stool samples obtained on days-of-life 12 and 30 were also negative for viral RNA.

From Canada, Yudin et al. [38] reported a 33-year-old pregnant woman who was admitted to the hospital at 31 weeks gestation with a fever, dry cough, and abnormal chest radiograph demonstrating patchy infiltrates. She had acquired SARS from contact with an infected family member. Following a 21-day stay in the hospital, during which she did not require ventilatory support, her convalescent antibody titers were positive for coronavirus infection. She had a normal labor and delivery and her newborn girl had no evidence of infection.

In a study of 5 liveborn neonates who were delivered to women infected with SARS-CoV during the Hong Kong epidemic, results from multiple tests—including serial RT-PCR assays, viral culture, and paired neonatal serological titers—were negative for SARS-CoV [39]. None of the 5 neonates developed any clinical signs or symptoms of respiratory infection or compromise. 

Fortunately, there were no cases of vertical transmission identified among pregnant women infected with SARS-CoV during the 2002–2003 Asian epidemic [27,30,31,39,40], and with the exception of a small cluster of cases that recurred in late 2003, no new cases of SARS have occurred.

## 6. Placental Pathology of SARS

In the only reported study of the placental pathology of mothers with SARS, Ng et al. [41] reported the findings from 7 pregnant women infected with SARS-CoV. In the case of 2 women who were convalescing from SARS-CoV infection during the 1st trimester of pregnancy, the placentas were found to be normal. Three placentas were delivered from pregnancies in which the mothers had acute SARS-CoV infection—these were abnormal and demonstrated increased subchorionic and intervillous fibrin, a finding that can be associated with abnormal maternal blood flow to the placenta. In the placentas of 2 women who were convalescing from SARS-CoV infection in the 3rd trimester of pregnancy the placentas were highly abnormal. They showed extensive fetal thrombotic vasculopathy with areas of avascular chorionic villi—chronic findings of fetal vascular malperfusion. These 2 pregnancies also were complicated by oligohydramnios and had poor obstetrical outcomes—both infants had developed IUGR. It is interesting that villitis, the microscopic finding of inflammation of the chorionic villi that is the histologic hallmark of many maternal hematogenous infections that are transmitted through the placenta to the fetus, was not identified in any of these placentas.

## 7. Safe Management of Pregnant Women with SARS

Similar to other coronavirus infections, SARS-CoV is easily spread from person-to-person via respiratory droplets and secretions as well as through nosocomial contacts [42,43]. In addition to transmission of SARS-CoV through natural aerosols from infected patients, it was found that in Hong Kong the SARS-CoV could also be transmitted by mechanical aerosols [44]. Environmental factors had an important role when it was discovered that during the Amoy Gardens housing estate outbreak as many as two-thirds of infected persons had diarrhea, SARS-CoV was excreted in their stools, and that aerosols arising from the flushing of toilets could transmit the virus [44]. Healthcare facilities were also an important source of new SARS infections during the 2002–2003 epidemic, and healthcare workers were also at high risk for acquiring the infection. 

In order to address the safety issues for the obstetrical management and delivery of pregnant women with SARS, guidelines were prepared by the Canadian Task Force on Preventive Health Care and the Society of Obstetricians and Gynaecologists of Canada [45]. These recommendations include:

1. “All hospitals should have infection control systems in place to ensure that alerts regarding changes in exposure risk factors for SARS or other potentially serious communicable diseases are conveyed promptly to clinical units, including the labour and delivery unit.
2. At times of SARS outbreaks, all pregnant patients being assessed or admitted to the hospital should be screened for symptoms of and risk factors for SARS.
3. Upon arrival in the labour triage unit, pregnant patients with suspected and probable SARS should be placed in a negative pressure isolation room with at least 6 air exchanges per hour. All labour and delivery units caring for suspected and probable SARS should have available at least one room in which patients can safely labour and deliver while in need of airborne isolation.
4. If possible, labour and delivery (including operative delivery or Caesarean section) should be managed in a designated negative pressure isolation room, by designated personnel with specialized infection control preparation and protective gear.
5. Either regional or general anaesthesia may be appropriate for delivery of patients with SARS.
6. Neonates of mothers with SARS should be isolated in a designated unit until the infant has been well for 10 days, or until the mother’s period of isolation is complete. The mother should not breastfeed during this period.
7. A multidisciplinary team, consisting of obstetricians, nurses, pediatricians, infection control specialists, respiratory therapists, and anaesthesiologists, should be identified in each unit and be responsible for the unit organization and implementation of SARS management protocols.
8. Staff caring for pregnant SARS patients should not care for other pregnant patients. Staff caring for pregnant SARS patients should be actively monitored for fever and other symptoms of SARS. Such individuals should not work in the presence of any SARS symptoms within 10 days of exposure to a SARS patient.
9. All health care personnel, trainees, and support staff should be trained in infection control management and containment to prevent spread of the SARS virus.
10. Regional health authorities in conjunction with hospital staff should consider designating specific facilities or health care units, including primary, secondary, or tertiary health care centers, to care for patients with SARS or similar illnesses.”

## 8. Middle East Respiratory Syndrome (MERS)

Middle East respiratory syndrome (MERS) was first reported in September 2012 in Saudi Arabia, following isolation of MERS-CoV from a male patient who died months earlier from severe pneumonia and multiple organ failure [1]. In the 8 years since then, there have been more than 2494 confirmed cases of MERS resulting in upwards of 858 deaths globally [46]. While 27 countries have reported cases of MERS, approximately 80% of confirmed cases originated in Saudi Arabia [47]. To date, all known cases of MERS can be linked to travel or residence in countries along the Arabian Peninsula—that is, Bahrain; Iraq; Iran; Israel, the West Bank, and Gaza; Jordan; Kuwait; Lebanon; Oman; Qatar, Saudi Arabia; Syria; the United Arab Emirates (UAE); and Yemen [48]. The largest documented outbreak outside of this region occurred in 2015 in the Republic of Korea, in which 186 infections occurred, resulting in 38 deaths [49]. The index case in this outbreak reportedly returned from the Arabian Peninsula just prior to onset of illness [50].

MERS-CoV is characterized by sporadic zoonotic transmission events as well as spread between infected patients and close contacts (i.e., intra-familial transmission) [51]. Nosocomial outbreaks in health care settings—the result of poor infection control and prevention—are widely recognized as the hallmark of MERS [1]. Superspreading events have been recorded in healthcare settings in Jordan, Al Hasa, Jeddah, Abu Dhabi and South Korea [47,52,53,54,55]. Like other coronaviruses, MERS-CoV can be spread through person-to-person contact, likely via infected respiratory secretions [48]. Transmission dynamics, however, are otherwise poorly understood [1]. Bats are believed to be the natural reservoir of MERS-CoV, and dromedary camels can have the virus and have been suggested as possible intermediary hosts as well as a source of infection to humans [2,56,57]. 

There are no clinical or serological reports of perinatal transmission of MERS, though vertical transmission has been reported for non-coronavirus respiratory viruses including influenza and respiratory syncytial virus (RSV) [58]. Researchers have not yet discovered ongoing transmission of MERS-CoV within communities outside of health care settings.

The clinical presentation of MERS varies from asymptomatic to severe pneumonia with acute respiratory distress syndrome (ARDS), septic shock, and multiple organ failure, often resulting in death. Most patients with MERS develop severe acute respiratory illness accompanied by fever, cough, and shortness of breath [50]. Progression to pneumonia is swift—usually within the first week —and at least one-third of patients also present with gastrointestinal symptoms [1]. MERS progresses much more rapidly to respiratory failure and has a higher case fatality rate than SARS [1]. Unlike SARS, however, infection with MERS-CoV is generally mild in healthy individuals but more severe in immunocompromised patients and people with underlying comorbidities [1]. The overall CFR of MERS is approximately 34.4% [46]. Most fatalities have been associated with pre-existing medical conditions like chronic lung disease, diabetes, and renal failure, as well as weakened immune systems [59], making such individuals high risk. As a result of the immunological changes that occur during pregnancy, women who are pregnant are included in this high-risk group. Pregnant women may develop severe disease and fatal maternal and/or fetal outcomes as a result of MERS-CoV infection; however, little is known of the pathophysiology of this infection during pregnancy.

## 9. MERS and Pregnancy

Limited data exists on the prevalence and clinical features of MERS during pregnancy, birth, and the postnatal period. It is likely, however, that the immunological changes that normally occur in pregnancy may alter susceptibility to the MERS-CoV and the severity of clinical illness [60]. Pregnant women infected with SARS-CoV, a related coronavirus, appear to have increased morbidity and mortality when compared to non-pregnant women, suggesting that MERS-CoV could also lead to severe clinical outcomes in pregnancy. To date, however, very few pregnancy-associated cases (*n* = 11) have been documented, with 91% having adverse clinical outcomes.

Between November 2012 and February 2016, there were 1308 cases of MERS reported by the Saudi Arabia Ministry of Health (MoH). Of these, 5 patients were pregnant, according to a retrospective study by Assiri et al. [47], and all resulted in adverse outcomes. Patient ages ranged from 27 to 34 years, with occurrence of exposure in either the 2nd or 3rd trimester. All 5 cases received intensive care. Two women died and there were 2 cases of perinatal death— 1 stillbirth and 1 neonatal death shortly after emergency cesarean section. These instances of severe maternal and perinatal outcomes are consistent with other reports of MERS-CoV infection in pregnant women, as well as outcomes associated with SARS-CoV infection. The authors of the retrospectives study concede that unreported cases of MERS in pregnancy are likely due to lack of routine pregnancy testing [47]. They conclude that pregnancy testing for women of reproductive age should be considered for those who test positive for MERS-CoV, to contribute to overall understanding of pathogenesis and epidemiological risk. Additionally, 2 of the 5 patients were healthcare workers, which corresponds with existing knowledge of higher risk of exposure to MERS-CoV in healthcare settings.

In a separate case report of MERS occurring in pregnancy, Alserehi et al. [58] described a 33-year-old critical care nurse who became infected during the 3rd trimester in the midst of a large hospital outbreak. In the days following hospital admission, she developed respiratory failure necessitating mechanical ventilation and administration of dexamethasone as prophylaxis for the fetus. Following an emergency cesarean section at 32 weeks gestation, she was transferred to the intensive care unit (ICU) and later recovered. The preterm but otherwise healthy infant was kept in the neonatal unit for observation and later released along with his mother. In contrast to other reported cases, this patient had a successful outcome, perhaps due to the timing of MERS-CoV exposure, her young age, the use of steroids, and differences in immune response.

Alfaraj et al. [61] described 2 cases of maternal infection with MERS-CoV at the Prince Mohammed Bin Abdulaziz Hospital (PMAH) in Saudi Arabia. Maternal infection in both cases was confirmed by nasopharyngeal swab testing by RT-PCR. One patient was a 29-year-old woman at 6 weeks gestation with no underlying medical conditions. The second patient, a 39-year-old at 24 weeks gestation, had several comorbidities, including end stage renal disease, hypertension, and hemodialysis. This woman presented to the hospital after contact with a MERS-CoV-infected person during an active outbreak. Both patients later tested negative for MERS-CoV and were subsequently discharged. The younger patient delivered a healthy, full-term infant. The status of the other delivery is unknown. Neither fetus was tested for MERS-CoV.

According to Payne et al. [62], epidemiologic investigation of the 2012 MERS outbreak in Zarqa, Jordan, revealed that a 2nd trimester stillbirth (5 months gestational age) had occurred as a result of maternal exposure to MERS-CoV. The mother experienced fever, fatigue, headache and cough, concurrently with vaginal bleeding and abdominal pain. On the 7th day of symptoms, she had a fetal death. The mother was confirmed to have antibody to MERS-CoV, and she self-reported having had unprotected contact with family members who later tested positive for the virus. This was the first documented occurrence of stillbirth during maternal infection with MERS-CoV.

On 24 November 2013, a 32-year-old pregnant woman in the United Arab Emirates (UAE) developed ARDS following admission to the ICU after suspected community-acquired pneumonia advanced to respiratory failure and hypotension [60]. Later that day, her baby was delivered by caesarean section and subsequent Apgar scores were within healthy range. The next day, RT-PCR evaluation revealed that the mother was positive for MERS-CoV. Despite rigorous intervention, including oral ribavirin-peginterferon-α therapy and ventilator support, the woman continued to deteriorate, developed septic shock, and died. While the outcome for this mother was fatal, Malik et al. noted that virus shedding ceased during therapy with ribavirin and peginterferon-α and radiographic evidence indicated clinical improvement before her death [58]. More research is needed to determine safety, efficacy, and dosage of these therapies in the general population but also in pregnant women. While few data exist on the effects of these treatments in pregnant humans, ribavirin is generally contraindicated during pregnancy [58].

Outside of the Middle East the only confirmed case of MERS in pregnancy occurred in 2015 in South Korea. Jeong et al. [49] reported that a 39-year-old patient was exposed during the 3rd trimester following contact with a patient having MERS. Despite abrupt vaginal bleeding and rupture of membranes, the patient recovered fully and delivered a healthy infant at 37 weeks and 5 days gestation. Subsequent testing of the infant’s blood did not detect any IgG, IgM, or IgA antibodies to MERS-CoV. 

The mean maternal age of the 11 confirmed maternal SARS cases described above was 33.2 years, with a mean gestational age of 26.3 weeks. The source of infection in 2 of the cases was attributed to contact with family members who tested positive for MERS-CoV, unknown in 3 cases, likely due to animal exposure in 1 case, and 6 were healthcare-associated (2 of these patients were healthcare workers). Six patients required intensive care and 3 died. Of those who died, 2 were exposed to MERS-CoV in the 3rd trimester, and 1 was exposed during the 2nd trimester. The infant death rate for all 11 cases was 27%. Fetal survival did not appear to correlate with the timing of maternal infection and gestational age; however, more data are needed to draw conclusions about this relationship. According to Alfaraj et al. [61], the CFR for the 11 infected women—also 27%—was not statistically different from the overall CFR of MERS in the general population (35%) (*P* = 0.75). Only 1 case resulted in both maternal and fetal death. 

Similar to SARS in pregnancy, more research is needed to understand the pathogenesis and epidemiology of MERS in pregnancy including the relationship between the timing of maternal infection, gestational age of the fetus, the effects of comorbid factors, and the occurrence of adverse outcomes. Few studies documented the presence of MERS-CoV antibodies in the umbilical cord or neonatal blood, making it difficult to assess perinatal transmission. As such, future studies should involve the collection of samples from relevant specimens including amniotic fluid, placenta, and umbilical cord [49].

## 10. MERS Prevention and Treatment

MERS prevention should be high priority for high-risk exposures such as healthcare workers, pregnant women and individuals working with camels, camel meat-milk processors and in abattoirs [57]. Since 2013, the Saudi Arabia MoH has recommended that pregnant women postpone travel to Saudi Arabia for the Hajj and Umrah [47]. To further reduce risk of exposure among pregnant women, additional measures such as avoiding contact with camels and sick persons—particularly in healthcare settings—are also recommended. Pregnant women who present with symptoms of pneumonia, influenza-like illness (ILI), or sepsis on the Arabian Peninsula may also benefit from MERS-CoV screening to expedite early diagnosis and improve disease management [60].

While multiple agents have been used to treat MERS, none have been tested in large clinical studies. Available data are limited to the use of combination therapies of interferon and other agents in case reports and case series [63]. A prospective or randomized study may prove difficult given the sporadic nature of MERS-CoV outbreaks.

Due to a gap in research on the treatment of MERS in pregnancy, there are no therapeutic options currently recommended for pregnant women [58]. Therapies under development and testing may be considered inappropriate for pregnant women due to the unknown potential for teratogenic effects. For example, during the 2003 SARS outbreak, ribavirin was administered to pregnant women with severe cases of the disease, but ribavirin therapy has been documented to increase the risk of teratogenic effects in newborns [58]. 

## 11. Other Coronaviruses and Pregnancy

The Alphacoronaviruses HCoV 229E and NL63, as well as the Betacoronaviruses HKU_1_ and OC43, can infect humans and cause the common cold. In order to investigate the potential maternal-fetal transmission of human coronaviruses during pregnancy, Gagneur et al. [64,65] evaluated 3 types of maternal-infant paired specimens that included maternal vaginal and respiratory specimens that were obtained during labor, as well as gastric samples from the newborn infants. These specimens were evaluated for the presence of HCoV 229E, OC-43, NL63 and HKU_1_ using RT-PCR methodology. Between the period from July 2003 to August 2005 the authors examined 159 mother-infant dyads. Human coronaviruses were identified in 12 samples (HCoV 229E: 11; HKU_1_: 1) from 7 mother-child pairs. In 3 mother-infant dyads only maternal respiratory samples were positive; in 2 other pairs all 3 of the samples tested positive for human coronavirus; in 1 case only the maternal vaginal and newborn gastric samples were positive; and in another case the maternal vaginal sample alone was positive. There were no signs of clinical infection in any of the 3 neonates that had positive gastric samples for human coronavirus. 

## 12. Participation of Pregnant Women in the Development of a Coronavirus Vaccine

It is beyond the scope of this communication to discuss the various technical challenges inherent in developing a safe and efficacious vaccine for coronavirus infections in humans. There are clearly challenges to this endeavor—protective antibodies to coronaviruses are not long-lasting, tissue damage has been reported to occur as a result of exposure to SARS-CoV, development of animal models that closely resemble human infection are limited, and the extensive time and expense necessary to perform clinical trials in humans, to name a few [66,67,68].

It is vitally important that pregnant women be considered in the design, clinical trial, and implementation of vaccine candidates for 2019-nCoV. In examining the history of vaccine design, it is clear that the needs of pregnant women have rarely been prioritized in either the preclinical development or the clinical trial phases of production. Today, pregnant women are usually excluded from experimental trial of drugs and vaccines that do not target obstetric conditions [69]. Excluding pregnant women and their infants from participation in vaccine development and implementation undermines ethical principles of justice—fairness, equity, and maximization of benefit—and potentially places their health at risk during outbreaks and other health emergencies [69,70,71].

On 23 January 2020 the Coalition for Epidemic Preparedness Innovations (CEPI) announced three programs to develop a vaccine against the novel Wuhan coronavirus. The Chief Executive Officer of CEPI, Richard Hatchett, said [72]:

“Given the rapid global spread of the nCoV-2019 virus the world needs to act quickly and in unity to tackle this disease. Our intention with this work is to leverage our work on the MERS coronavirus and rapid response platforms to speed up vaccine development.”

The novel coronavirus is the first epidemic disease to emerge since the formation of CEPI in Davos in 2017. CEPI was created with the express intent to enable speedy research and development of vaccines against emerging pathogens. In May 2017, WHO released the Target Product Profile (TPP) for MERS-CoV vaccines, following the prioritization of MERS-CoV as one of eight priority pathogens for prevention of epidemics [73]. CEPI and partners aim to use existing platforms—that is, the existing “backbone” that can be adapted for use against new pathogens—that are currently in preclinical development for MERS-CoV vaccine candidates. Following the WHO declaration on 30 January that the current 2019-nCoV outbreak is a public health emergency of international concern (PHEIC), global health organizations and researchers will be further mobilized—bolstered by new mechanisms for action and greater resources—to stop the spread of disease. 

A critical question that must be answered at this stage—with a clear view of the potential deleterious effects of a new coronavirus in pregnancy—is will maternal immunization be a priority in research and development? As of the PHEIC declaration, 12 groups have announced that they are developing new vaccines against 2019-nCoV and seven others announced initiatives to develop new therapies [74]. Safe testing of experimental vaccines in a pregnant population is difficult and, as a result, vaccines are not typically developed with pregnant women in mind. To date, very few clinical trials for vaccines have proactively included pregnant women [75], and the exclusion of pregnant and lactating women from receiving the rVSV-ZEBOV vaccine through 3 Ebola virus epidemics serves as a recent example [69,70,71]. Given the potential severity in pregnancy, as demonstrated by this review of maternal infections of SARS and MERS, women who are pregnant should be considered a priority population in all efforts to prepare for and prevent infection by novel coronaviruses.

## 13. Current Status of 2019-nCoV (SARS-CoV-2) Infection of Pregnant Women and Neonates

On 5 February 2020 it was reported by multiple media outlets that a newborn infant delivered during the epidemic in Wuhan had tested positive for 2019-nCoV at the Wuhan Children’s Hospital in Hubei Province 30 hours following its birth. According to the official Xinhua news agency, the infant was delivered on 2 February to a mother who had tested positive for the virus. Reports have stated that the infant had stable vital signs, no fever or cough, but had shortness of breath together with abnormal chest radiographs and abnormalities of liver function [76,77,78]. Dr. Zeng Lingkong, Chief Physician at the Neonatal Medicine Department of the hospital, said [78],

“This reminds us to pay attention to mother-to-child being a possible route of coronavirus transmission”

The hospital also provided information about a previous case of a baby that had been delivered on 13 January 2020. Following its birth, the infant’s nanny was diagnosed with 2019-nCoV, and the mother was diagnosed days later [76]. On 29 January the baby began to develop symptoms. According to Dr. Zeng Lingkong [76],

“Whether it was the baby’s nanny who passed the virus to the mother who passed it to the baby, we cannot be sure at the moment. But we can confirm that the baby was in close contact with patients infected with the new coronavirus, which says newborns can also be infected”

In considering whether these and future cases of neonatal infection are acquired prior to delivery, it is important to remember that newborn infants can acquire an infection in other ways beyond intrauterine maternal-fetal transmission. In some cases, viral infection can be acquired when the infant passes through the birth canal during a vaginal delivery or through post-partum breast feeding, although these mechanisms would be highly unusual for a respiratory virus. Neonatal infection from respiratory viruses can occur after delivery through such mechanisms as inhalation of the agent through aerosols produced by coughing from the mother, relatives or healthcare workers or other sources in the hospital environment. Based upon past experience with pregnant women who developed MERS and SARS, and realizing that the numbers are limited, there has never been confirmed intrauterine coronavirus transmission from mother to fetus. Discussing the most recent baby to be diagnosed with the 2019-nCoV infection, Dr. Stephen Morse, an epidemiologist at the Mailman School of Public Health at Columbia University stated [77],

“It’s more likely that the baby contracted the virus from the hospital environment, the same way healthcare workers get infected by the patients they treat,”

“It’s quite possible that the baby picked it up very conventionally—by inhaling virus droplets that came from the mother coughing.”

And according to Dr. Paul Hunter, Professor of Medicine at the University of East Anglia [79],

“As far as I am aware there is currently no evidence that the novel coronavirus can be transmitted in the womb. When a baby is born vaginally it is exposed to the mother’s gut microbiome, therefore if a baby does get infected with coronavirus a few days after birth we currently cannot tell if the baby was infected in the womb or during birth.”

## 14. Conclusions

There is limited knowledge regarding coronavirus infections that occur during pregnancy—what is known has, for the most part, been the result of epidemics resulting from two different diseases, SARS and MERS. These previous experiences with coronavirus infections in pregnancy indicates that these agents are capable of causing adverse clinical outcomes including life-threatening maternal disease that in some cases requires hospitalization, intensive care and ventilatory support. Both of these coronaviruses can result in maternal death in a small but significant number of cases, but the specific risk factors for a fatal outcome during pregnancy have not been clarified. Coronaviruses can also result in adverse outcomes for the fetus and infant including intrauterine growth restriction, preterm delivery, admission to the ICU, spontaneous abortion and perinatal death. Unlike some viral infections, notably Ebola virus [70] and Zika virus [80], the likelihood of intrauterine maternal-fetal transmission of coronaviruses is low—there have been no documented cases of vertical transmission occurring with either SARS or MERS. It remains to be seen during the current Wuhan 2019-nCoV epidemic how this newly-emergent coronavirus affects pregnant women and their infants, as well as which factors may modulate obstetrical disease and outcomes including the timing of maternal coronavirus exposure by gestational age, the effects of medications or other treatment regimens, differences in host immune responses, occurrence of coexisting medical and obstetrical conditions, and other covariables. However, pregnant women should be considered to be at high risk for developing severe infection during this current outbreak of 2019-nCoV. Additional clinical research on the treatment of SARS, MERS, and the new coronavirus 2019-nCoV is necessary if we are to understand the potential risks and benefits of novel therapies and new vaccines in pregnancy. This research will be critical in improving the care, and even saving the lives, of pregnant women in the current as well as future outbreaks.
